# Impact of knowledge management capabilities on new product development performance through mediating role of organizational agility and moderating role of business model innovation

**DOI:** 10.3389/fpsyg.2022.950054

**Published:** 2022-07-28

**Authors:** Hisham Idrees, Josef Hynek, Jin Xu, Ahsan Akbar, Samrena Jabeen

**Affiliations:** ^1^School of Economics and Management, Southwest Jiaotong University, Chengdu, China; ^2^Department of Informatics and Quantitative Methods, Faculty of Informatics and Management, University of Hradec Kralove, Hradec Králové, Czechia; ^3^School of Economics and Management, Southwest Jiaotong University, Chengdu, China; ^4^International Business School, Guangzhou City University of Technology, Guangzhou, China; ^5^Faculty of Business Management, University of Bahrain, Zallaq, Bahrain

**Keywords:** knowledge management capabilities, new product development, organizational agility, business model innovation, Chinese automobile sector

## Abstract

In several studies, knowledge is witnessed as one of the foundations of long-term competitive edge and is also a basic source of new product development (NDP) performance. The aim of this study is to investigate the role of knowledge management capabilities (KMC) in new product development performance with the mediating role of organizational agility. Additionally, this study also intends to examine the moderating role of business model innovation on the relationship of KMC with organizational agility. This study was conducted on the Chinese automobile sector, and the NPD project managers, supervisors, and engineers of the sector were respondents of this study. A survey questionnaire was used to collect the data from 201 respondents, and data were analyzed using the Smart PLS 3 software. The findings of this research, although limited to the automobile industries, indicate that knowledge sharing and knowledge application have significant and positive effects on the development of new products. Organizational agility significantly mediates the relationship of KMC with NPD. The results found that business model innovation has a significant moderating role in the relationship between KMC and organizational agility. Moreover, the results of this study will assist the managers in developing a modern competitive business environment by implicating KMC in the process of NPD. Lastly, organizations may improve the sustainability of their product and their overall performance by using organizational agility and modern ways of value delivery.

## Introduction

In a contemporary business environment of higher complexity and uncertainty, the organization always faces issues of awareness that what kind of information, competencies, and expertise are essential for an organization to take widespread advantage of available opportunities (Husnain et al., 2021). In addition to these competencies, an organization must possess unique features; therefore, competitors find it difficult or are unable to imitate them while operating in the same market and industry (Sousa and Rocha, 2019; Sohail et al., 2020). In the situation of intense competition with a higher need for a new market, it is difficult for a new product to capture the market successfully because of the high failure rate, which is ~40% of new product development (NPD) (Haider and Tehseen, 2022). A large number of researchers, practitioners, as well as managers associated this failure with less awareness of continuous changes in the sector (Jiao et al., 2019; Kafetzopoulos et al., 2019). In this regard, Allameh et al. (2017) recommended that organizations must keenly and constantly consider changes that are taking place in the market and should design their processes according to the market demands. For the NPD effectively, organizations are necessarily required to recognize the needs and wants of the market for obtaining accurate and up-to-date information. Moreover, organizations should recognize what kind of competencies are required to avail the market opportunities effectively (Shahzad et al., 2021).

While developing products innovatively, the primary role of knowledge management (KM) is to develop, introduce, and sustain a competitive advantage with the help of appropriate knowledge application and collaborative operations (Haider, 2019). However, Acharya et al. (2022) concluded that introducing and sustaining an innovative product is extremely complex because of reasons, such as varying demands of customers, the intensity of competition in the market, and quick technological advancements. It has become difficult for organizations to achieve sustained innovation and competitive advantage internally. Therefore, some of the large organizations, like Xerox and Hitachi, had made collaborations outside the organizations to achieve sustained competitive advantage and innovation (Lee and Choi, 2021). Such collaborations may facilitate the KM or result out from the firm's pursuit to use new knowledge. In a collaborative relationship, an organization has access to the operations and processes that other organizations use, and these can be incorporated into various settings. Information and competencies acquired through collaboration are regarded as efficient and effective sources of successful innovation. Besides, appropriate information management and its application reduce complexity while developing a new product as knowledge is regarded as a resource that has significant importance in the innovation process (Pitt and MacVaugh, 2008). In product development, innovation entirely depends upon the availability of information; therefore, the complex phenomenon of explosion of richness and availability of knowledge is required to be considered and managed appropriately (Haider and Kayani, 2020). Marion and Fixson (2021) indicated that an increased amount of information readily available for business organizations creates more complexity regarding designing and controlling while developing a new product. However, this uncertainty and complexity can be reduced by managing knowledge with the help of a strategic nature of knowledge-intensive business units.

Knowledge management (KM) capabilities are necessary and exceptional for an organization. Organizations are required to decide what kind of knowledge is required, how to attain it, and its application mechanism to reap effective and efficient results in the shape of innovation while developing a new product that ultimately will attain a sustained competitive edge (Cerchione et al., 2016; Attia and Salama, 2018; Haider et al., 2021). Arora and Ratnasiri (2015) focused on the importance of knowledge with regard to economic performance in the Asian economy. In the international rivalry, knowledge is regarded as a key resource for small and medium enterprises (SMEs). It is considered as a significant contributing factor for the success of SMEs and certainly an important factor of sustainable competitiveness. According to Acharya et al. (2022), many businesses are shifting to knowledge specialties. In relation to these firms, KM is an innovative management method that benefits them in theory application, as well as in practice (Shahzad et al., 2021).

In the modern business environment, it is necessary that there should be an established organizational mechanism for managing information that may smoothly distribute information among employees of the organization and remove barriers to the generation and acquiring of knowledge (Balodi, 2014). The cultural component of an organization plays a significant role because it is the culture that inspires organizational employees to search for innovative information and idea generation. Moreover, the technological factor is also significant in the firm because it provides improved means of communication and helps discover innovative knowledge (Imran et al., 2021). Furthermore, organizational agility (OA) is an important ability that helps organizations to capture and utilize emerging opportunities rapidly by adjusting the activities of their ongoing new product development projects (Haider et al., 2021). Organizational agility refers to the ability of an organization of recognizing and responding to market variations (Rafi et al., 2021). Organizational agility enables firms to assess market information timely and appropriately in the decision-making regarding product development (Najafi-Tavani et al., 2018). It also facilitates organizations in the application and execution of innovative strategies developed on the basis of acquired knowledge (Chakravarty et al., 2013). Illustratively, Apple assessed the market potential of smart wearables, quickly focused on the development of smartwatches, and released it within 2 years, which captured 75.5% of the smartwatch market's share globally (Cai et al., 2019). On the other hand, attaining this kind of agility is not easy for most of the business organizations (Lee and Choi, 2021). However, it is necessary to understand how organizational agility contributes to the development of a new product.

Organizations can create innovation in all aspects of business by utilizing available opportunities, creating value for customers, and by providing better service delivery by addressing issues of sustainability (França et al., 2017). Osterwalder and Pigneur (2010) described the business model as the foundation that how a business unit generates, distributes, and captures value. Business model canvas (BMC) has become a *de-facto* benchmark for the development of business models (Rachinger et al., 2018). Additionally, Geissdoerfer et al. (2018) argued that the business model is now an attention-seeking topic of research for scholars. The innovative business model is regarded as an imperative factor for sustainable organizations (Visnjic et al., 2016). Nevertheless, a major hurdle prevails that the design of operating business model traditionally fails to integrate the dimension of sustainability (Upward and Jones, 2016). Hence, the extent of sustainability is generally not appropriately comprehended because of insufficient planning, narrow scope of operations, and low competence of human resources in integrating employees and systematic undertakings in sustainable manners (Rohrbeck and Schwarz, 2013). Ultimately, the opportunity for innovation and sustainability during business-value generation processes is wasted.

The purpose of this research study is to offer a profound, thoughtful understanding of the effect of knowledge management capabilities in the development of new products by mediating the role of organizational agility and moderating the role of business model innovation (BMI). The research focuses on automobile parts manufacturing firms in Guangdong China, where they are offering innovative and unique products in the market. Automobiles parts manufacturers are such organizations in which the issues of innovation, KM, and swiftness to respond are critical elements. Therefore, improving KMC was focused on significantly influencing the business capacity to create new products. In practice, this study significantly provides a deeper insight and better understanding to automobile sector managers in leading and governing innovation and avoiding failures of being left behind.

## Literature review

### Theoretical background

In organizations where various kinds of knowledge capabilities are required for innovation, knowledge management is regarded as a key success factor and plays a vital role in achieving competitive advantage (Attia and Salama, 2018; Garcia-Perez et al., 2020). This study introduces KM by describing its capabilities and influence on creating new products and services. An extensive study of the relevant literature suggests a theoretical model incorporating various factors. In maintaining competitiveness, organizations consider KM as an optimal instrument. Previous studies explored that KM is the formation and acquisition of knowledge by the stockholders of an organization intrinsically, as well extrinsically to disseminate, integrate and store in the three phases that are applied to establish effective knowledge resources and benefits for the generation of higher profit (Nonaka and Takeuchi, 1996). Hence, with reference to the various research studies, this study establishes three dynamic processes with regard to the content and nature of KM mechanisms by categorizing them into (1) “Knowledge creation and acquisition”, (2) “Knowledge diffusion and integration”, and (3) “Knowledge storage and Application”. The above-mentioned three dynamic processes are applied to explore KM mechanisms as research variables in the industry.

### Knowledge management capabilities

Rafi et al. (2021) defined KM as the availability and formation of expertise, information, and competencies that shape new capabilities, result in better performance, motivate innovation, and generate higher value for customers. Visionary managers always consider and focus on the need of developing and utilizing knowledge for the prosperity of the organization (Haddad and Ribière, 2007). Knowledge can be described as understanding the association, situation, phenomena, concepts, and procedures of a prevailing problem or domain (Naqshbandi and Jasimuddin, 2018). With respect to the competitive advantage, knowledge has vital and increasing significance in organizations. Knowledge of the contemporary situation is regarded as the foundation of innovations in organizations. Hence, organizations are intensely required to recognize innovative knowledge for innovation initiation (Garcia-Perez et al., 2020). Knowledge is broadly categorized into two kinds that are explicit and tacit knowledge. The explicit form of knowledge is found in textbooks, research articles, and guiding manuals; however, tacit knowledge is difficult to contextualize (Zahoor et al., 2022). Defining tacit knowledge, Kikoski and Kikoski (2004) said, “What are unsaid and unexpressed could be the reservoirs of tacit knowledge”. So, it is difficult to acquire, identify, and communicate tacit knowledge for an organization. Cooperation among people is needed for the success of KM. Davenport (1996) further added that KM meant gathering information and communicating to the individuals in demand. Collective activities that favorably enhance the resource of organizational knowledge, including gaining, formation, application, and communication of knowledge, are called “knowledge management”.

It is essential for organizations to have the ability of recognizing and leveraging new knowledge for competing in the market and attaining competitiveness (Gold et al., 2001). Thus, an important concern of the organizations arises is how they can effectively allocate resources while developing new products and services that create a competitive edge for the organizations over their rivals. For this reason, a business is required to incorporate knowledge in the way of value creation from the intangible resources of an organization (Löfsten, 2014). Management of knowledge comprises of various elements, including identifying, recognizing, generating, applying, communicating, and storing it (Liu and Tsai, 2007; Martinez-Conesa et al., 2017) concluded that activities of knowledge management are decisive for innovation application. Consequently, the organizational role is not merely limited to the acquisition of competences. Moreover, organizations are essentially required to develop organizational knowledge as it is regarded as a resource and a foundation of competitiveness and differentiation in the organization. In prior research, Costa and Monteiro (2016) concluded that KM has a proven impact on innovation with respect to the development of product and service because it initiates innovations.

Furthermore, Zaim et al. (2019) discussed that irrespective of the knowledge generation or innovation, the knowledge wave started was when people involved in sharing knowledge among groups or persons. Chang et al. (2017) explained that many business organizations recognized creativity as the key to competitive advantages, and knowledge is the key to continuous creativity. Creative knowledge has become a topic of wider attention in research. Based on the extensive literature, this research considers gaining, creation, storage, and diffusion of knowledge as the primary constructs of “KM capabilities.” Past studies by Chang et al. (2017) and Jasimuddin and Naqshbandi (2019) discussed two major kinds of knowledge management capabilities. The first kind of capabilities deals with the structure of KM because it provides a framework to the organization that enables the knowledge flow within the organization, as well as in the external context (Kim et al., 2013). These kinds of capabilities are called “knowledge infrastructure capabilities”. The second kind of capabilities are associated with the dynamic activities of KM by recognizing dynamic variations in the environment and making the organization able to adopt capabilities that may effectively deal with these dynamic changes (Lee and Choi, 2021). These kinds of capabilities are called “knowledge-based dynamic capabilities”.

For a sustained competitive advantage, organizations must appropriately practice the activities of generating, acquiring, storing, communicating, and implementing knowledge for problem-solving and exploring available opportunities (Shahzad et al., 2021). However, effective KMC involves an understanding of connections that prevails between KMC processes, including acquiring, generating, storing, communicating, and implementing the knowledge (Seleim and Khalil, 2011). Businesses with a higher level of KMC practices enable them to develop a learning environment that expands their competencies of reducing redundancy, responding efficiently to market variations, and emerging creative and innovative ideas (Bresnen et al., 2003). The decision-making quality of a business depends upon gaining, communicating, and applying knowledge among individuals and groups in the organization. In KMC, knowledge acquisition and application are rudimentary and the main goal of the knowledge management process (Gold et al., 2001). Organizational culture, information technology, and organizational structure make organizational infrastructure capability contribute to knowledge sharing (Gharakhani and Mousakhani, 2012). So, for a more concise understanding and in line with previous scholarly work, this research takes into account and focuses on the three key capabilities, i.e., acquiring, distributing, or sharing knowledge, and the usage or application of knowledge.

#### Knowledge acquisition

Acquisition of knowledge is a part of KM that is defined as “the process of critically managing knowledge to meet existing needs, to identify, and exploit existing and acquired knowledge assets and to develop new opportunities” (McAdam and Reid, 2001). It is also described as the process of obtaining knowledge. Acquired knowledge may be of a tacit nature or of an explicit kind or a combination of both kinds. The acquisition takes place with the contribution of individual, interactive tasks, technological applications, asset utilization, and human resources deployment in a specific setting (Tsoukas and Vladimirou, 2001). Many researchers suggested that knowledge management in an organization is an ongoing process that results in knowledge acquisition (Harsh, 2009). Two primary sources of knowledge gathering are exploring entirely new knowledge and creating new knowledge from the existing database through interaction among individuals and business counterparts (Harsh, 2009; Dost et al., 2019). Numerous scholars focused that knowledge acquisition is critical when an organization collaborates with others (Martinez-Conesa et al., 2017; Shahzad et al., 2021). External channels of a firm are significant sources of acquiring knowledge. Codified and non-codified means of external information frequently generate valued information and provide innovative knowledge (Assimakopoulos and Yan, 2006). Though acquiring knowledge from external sources is not an easy task for a business, it provides information of greater value. External sources of acquiring knowledge include gathering public information from internet sources, books, personal networks, and professional associations (Choi et al., 2010).

#### Knowledge sharing

The purpose behind sharing knowledge is to generate new knowledge by collaborating on existing information or appropriately expanding existing knowledge (Rehman et al., 2021). In literature, knowledge sharing is defined as a culture of social contact that contains sharing of knowledge, expertise, and skills among employees working in an organization or department (Chang et al., 2017). It also contains a combination of shared understandings regarding access of employees to the related data for the development and application of networks inside the business (Akhtar et al., 2022). Knowledge sharing is associated with the common beliefs or behavioral practices about sharing of learning between various persons or departments working in a concern (Moorman and Miner, 1998). It refers to the persons, groups, and entities that share and acquire information from each other. In addition, networks of personal, as well as organizational levels, are important for knowledge accessibility. Personal or virtual networks help in knowledge sharing, and without these networks, it is hard to access knowledge. Furthermore, networks are possible to maintain with the help of face-to-face meetings officially or casually. For the success of an organization, sharing of knowledge is a critical factor (Davenport, 1996). The major hurdle regarding knowledge sharing is to convince, compel, or command people within the organization for sharing information they possessed (Jiao et al., 2019). With respect to the organizations, sharing of knowledge include acquiring, arranging, recycling, and communicating experience-based knowledge that prevails inside the organization and sharing that information with others inside the business. Hence, knowledge sharing enables a business to create opportunities for the enhancement of the organizational ability to deal with the requirements of the market (Li et al., 2022), provide an effective solution, and helps in attaining competitive advantage in the long run (McAdam and Reid, 2001).

#### Knowledge application

Knowledge application is another significant dimension of the process of KM. Therefore, Husnain et al. (2021) stated that the value of knowledge resourced is recognized at the time of developing new products, providing services, or when these products or services are transacted for value. It is regarded as the focal component of the KM procedure (Haider and Kayani, 2020). According to the knowledge-based model, the worth of personal, as well as organizational knowledge, mainly resides in the application of knowledge due to stickiness of it (Grant, 1996). Some of the researchers defined the application of knowledge as the implementation and utilization of knowledge in the value-adding process of an organization. It also comprises the placement of knowledge in the expansion or creation of organizational ability (Song et al., 2005). In addition, it includes identifying, integrating, and implementing knowledge in the products and operations of an organization. However, “knowledge application capability” can be described as the competence of employees to apply information for the development of problem-solving frameworks and coping with the prevailing issues that business is facing during the process of NPD (Sarin and McDermott, 2003). With the effective application of knowledge, some mistakes are also expected at the individual employee level; however, it may improve the efficiency of employees and decrease redundancy (Choi et al., 2010).

## Hypotheses development

### Knowledge management capabilities and new product development

Song et al. (2005) stated four factors for assessment of relative success in NPD: quality of the newly introduced product as compared to the product of competitors, sales volume of newly introduced product in comparison to that of competitors, rate of return of newly introduced product in comparison to that of rivals, and the ratio of success regarding new products introduced in the market with the estimated return targets. In addition, Calantone et al. (2002) considered the investment ratio, the growth rate of investment, the sales ratio, share of market, and its growth rate as the evaluating factors of NPD performance. Besides, Hopkins (1981) applied these five elements for the measurement of NPD performance, including target assessment, financial evaluation, contribution of new products in total sales, market share of successful new products, and customer satisfaction regarding NPD. Generally, organizations introduce new products according to the expectations of the customers, and they try their best to apply their capabilities and strengths for the creation of valuable products. For this reason, KM is the key tool that enables organizations to apply their acquired knowledge in the form of appropriate effort (Haider and Kayani, 2020). It also helps in comparing the capabilities of business with other counterparts and molding their operations to attain competitiveness (Cepeda-Carrion et al., 2017). By realizing the importance of KM, organizations are able to attain superior competence in relation to all of their competitors.

Consequently, organizations enhance their productivity by opting this and it will help for the introduction of innovative features and designs in products that potentially will be recognized by the targeted customers as it addresses the needs and demands of customers. It is also regarded as an important aspect of market orientation in confirming the success of a newly introduced product (Donate and de Pablo, 2015). In line with the above arguments, organizations with appropriate knowledge management always look for superior quality offerings and technologies that are necessary for the assimilation of quality elements. Actually, fulfilling the criteria to maintain standards is a demand of customers. Hence, stability in the association of information storage and value generation for customers is a primary theory of every NPD organization looking for distinction (Tzokas et al., 2015). According to the knowledge-based view of the organization, how efficiently an organization develops its innovative competencies by exchanging various pieces of information among individuals and departments. It will certainly enhance coordination among knowledgeable persons. For the exploration of creative ideas, these kinds of interactions among team members are productive and essential that ultimately generate innovative knowledge for the successful offering of new products (De Clercq et al., 2015). Accordingly, the hypotheses proposed are as under:

Hypothesis 1: Knowledge acquisition is positively associated with new product development.
Hypothesis 2: Knowledge sharing is positively associated with new product development.
Hypothesis 3: Knowledge application is positively associated with new product development.

### Mediating role of organizational agility

In literature, researchers recognized that description of organizational adaption is hard due to variations, complexity, and instability (Kammerlander and Ganter, 2015). In an unstable and uncertain environment, dynamic alignment and management capability are key factors of success for an organization. Organizational agility (OA) is applied for the contextualization of a situation. Numerous scholars described “organizational agility”, but the explanations (Hatum and Pettigrew, 2006; Bernardes and Hanna, 2009) are more appropriate. Here, Teece et al. (2016) defined OA as the capability of a business to transform its assets into value for customers operating in volatile internal and external environments. Moreover, OA is also described as the responding ability of a firm in turbulent environments innovatively that also include unforeseen variations regarding technology and demand (Tallon and Pinsonneault, 2011). In addition, these turbulences produce opportunities and threats where businesses must discover innovative solutions to deal with the phenomenon. Furthermore, Bernardes and Hanna (2009) stated that agility is a strategy for managing unpredictable turbulence. It is the capacity of a business to deal with uncertain situations and is associated with the dynamic capabilities of business (Teece et al., 2016). For the development and enrichment of business agility, Shafer et al. (2001) suggested three steps, including initiation, adaptation, and distribution. First, initiation is concerned with the competence of business to avail opportunities and deal with the prevailing threats.

Second, adaptation is associated with the quickly responding capability of business regarding internal and external variations in the business environment. Last, delivery deals with the operational progress of business in effective and efficient manners. So, firms with agile ability proficiently, sustainably, and profitably operate in uncertain settings (Jacobs et al., 2011). Organizations operating in diverse industries adopted the concept of agility, including manufacturing concerns (Theyel and Hofmann, 2020), software businesses, supply chain networks (Mohammadi et al., 2019), and project management (Ahimbisibwe et al., 2017). However, the concept of OA is not explained in these industries uniformly (Koch and Schermuly, 2020). Illustratively, there are various methods to advance OA in the software industry, likewise scrum, software development learning, dynamic system development technique, and extreme programming (Conforto et al., 2014; Nicholls et al., 2015). In addition, a higher capability to reconfigure processes, including machining and workflow, to cope with the various demands of customers also increases organizational agility. From product development planning to launching a new product in the market, development teams deal with the various uncertainties in the NPD process. The application of pre-determined solutions in uncertain situations of the NPD process is highly risky (Wang, 2017). Dealing with uncertain situations with the application of agile abilities, development teams have more opportunities of controlling risk and generating new ideas where these ideas may be applied in the development procedure. Therefore, firms are required to consider the significance and rewards of adopting OA before the NPD. Accordingly, the hypotheses proposed are as under:

Hypothesis 4: Organizational agility is positively associated with new product development.
Hypothesis 5: Organizational agility mediates the relation between knowledge acquisition and new product development.
Hypothesis 6: Organizational agility mediates the relation between knowledge sharing and new product development.
Hypothesis 7: Organizational agility mediates the relation between knowledge application and new product development.

### Moderating the role of business model innovation

Generally, a business model (BM) is acknowledged as a framework in what way an organization generates and conveys value to customers and what apparatuses are applied to gain value (Teece et al., 2016). By applying the BM idea, a business defines its scope in terms of “what it does,” “what its offers,” and “how the offer is made” (Ritter and Lettl, 2018). In addition, business model innovation (BMI) is defined as the process of developing BM that may be new for that particular organization or new to the entire industry (Björkdahl and Holmén, 2013; Foss and Saebi, 2017). Moreover, BMI is elucidated as the outcome of an innovative move by substituting the existing BM in an organization or entirely modifying any BM carried by a business (Lindgardt et al., 2012). These changes may be in terms of the value chain or the value proposition with regard to the customers or partners in an organization (Matzler et al., 2013).

BMI is the modification in the existing operational designs of a business ranging from the focal firm and its customers, shareholders, suppliers, and rest of the stakeholders contributing to the value-creating process (Andreassen et al., 2018). Value creation components helping BMI are more varied, environment specific, and shortly explained than those associated with product innovation (Clauss et al., 2019). Additionally, Amit and Zott (2010) have the opinion that BMI-supported e-businesses generate value with the help of added innovation, complementarity, competence, and lock-in. The value creation process of incumbent in the “nondigital” industry particularly is a topic of greater interest for researchers (Amit and Zott, 2015). Similarly, the first contribution regarding that in what ways manufacturing business generates value. Particularly, the application of value for customers by transferring the model to the service industry is started to appear (Raja et al., 2013). Accordingly, the interaction between product development and BMI requires attention greatly. Nowadays, scholars are paying greater attention to the supporting role of BMI in enhancing value creation while developing a product (Desyllas and Sako, 2013). They also emphasized that firms must consider the way how BMI and product innovation are associated with each other (Gambardella and McGahan, 2010) In addition, BMI can enhance the influence of KMCs during new product development. Accordingly, the hypotheses proposed are as under:

Hypothesis 8: Business model innovation plays a moderating role in the association between knowledge acquisition with organizational agility.
Hypothesis 9: Business model innovation plays a moderating role in the association between knowledge sharing with organizational agility.
Hypothesis 10: Business model innovation plays a moderating role in the association between knowledge application with organizational agility.

## Research framework

**Figure 1:**
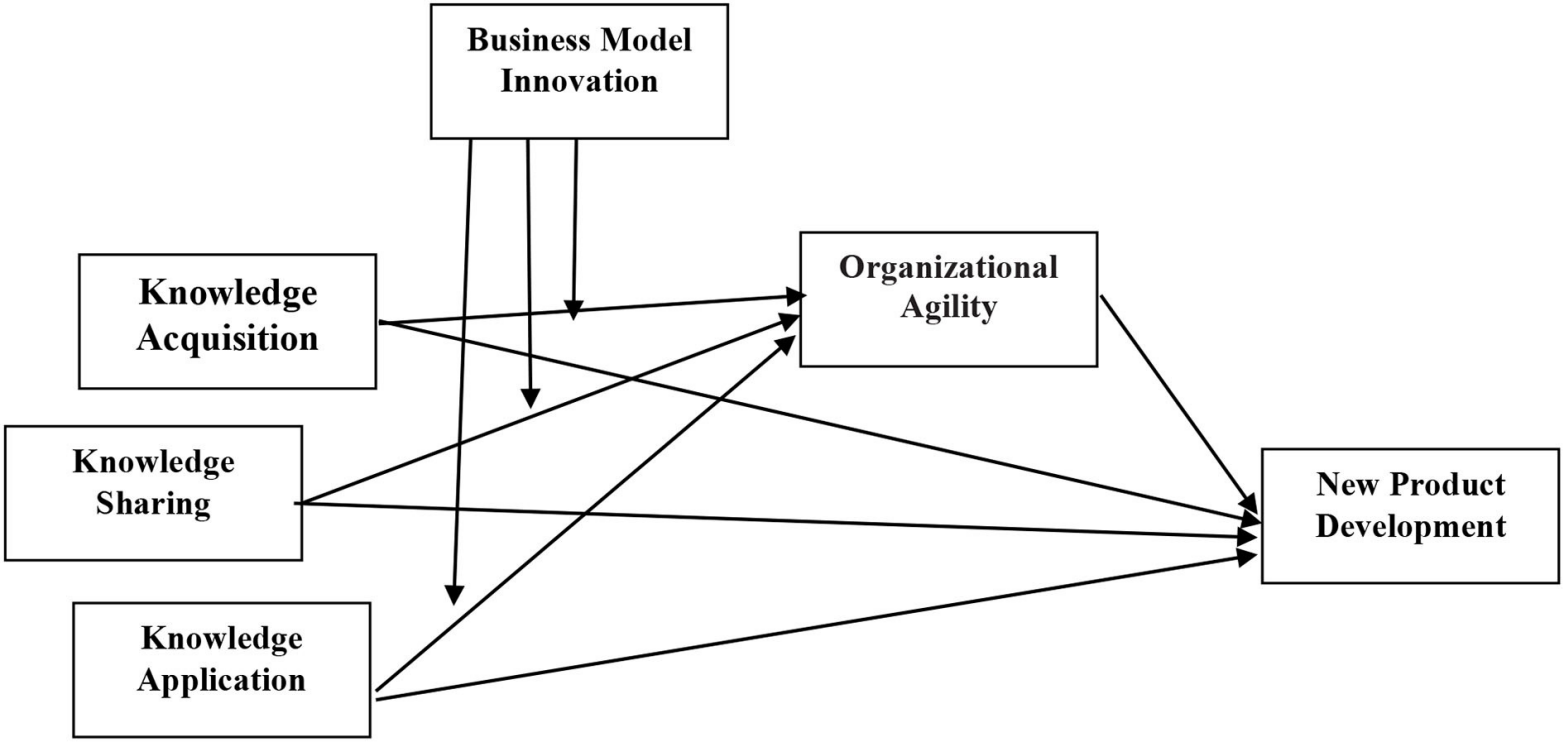
Conceptual model.

In accordance with the scholarly work of previous authors, this study takes into account the process capabilities factors and examines how they impact the new product development performance under circumstances where firms are able to adapt and modify their operational design in the process of creating value. Therefore, this study measures three knowledge management capabilities processes [knowledge acquisition (KA), knowledge application (KAP), and knowledge sharing (KS)] from the perspectives of Gold et al. (2001); Lin and Lee (2005), and Gharakhani and Mousakhani (2012) at the SME level on NPD performance (see Figure 1), but with a different approach and methodology.

## Research methodology

### Sampling and procedure

This study uses online questionnaires to collect data. The automobile parts manufacturing firms in Guangdong province were surveyed for this study as sustainability and going green, such as reduction in CO_2_ emission and light weight production, are key factors forcing firms to continually develop new products and consider new designs (Wellbrock et al., 2020). Moreover, China is the leading manufacturer of automobile parts, so it is impossible to collect data from the whole population, so we strategically selected five cities in Guangdong province. Because of resource and time constraints, convenience sampling was used to collect and analyze the data. 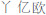 EqualOcean Company's data list was used to identify the main industry players in the automobile sector of the five cities in Guangdong province (Guangzhou, Shenzhen, Zhuhai, Foshan, and Dongguan). As part of the data collection programs, 100 firms with employees in the range of 300–1,500 were approached to participate in the study. Only 36 firms agreed to participate in the email questionnaire survey. Firms were solicited to identify their NPD managers, Team leaders, engineers, and supervisors with adequate knowledge of KM and NPD processes to participate in the study as they were more likely to present a large view of the firm's level NPD performance. Participants were informed that their responses would remain anonymous. Data were collected in 8 months, from October 2021 to May 2022. This study uses a cross-sectional approach and quantitative data techniques to establish links among the constructs of the study (Nardi, 2018).

Thirty-six agreed firms from the list of 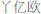 EqualOcean Company's data list were contacted through telephone calls and emails. The main challenge for this research was to collect face-to-face data because of COVID-19. Therefore, we requested each of the agreed firms to identify the key contact persons with adequate knowledge and can act as sources and help in the data collection process. In total, 360 questionnaires accompanying a cover letter with the full explanation of our detailed research objectives and declaring privacy and confidentiality were sent out to identified participants at their email addresses. To increase and improve our response rate not only follow-up calls and emails were used, but we also offered an incentive to share our aggregate survey findings with the informants who completed and returned their questionaries. According to the G^*^ power software, 153 participants are required for this study to reach a power of 0.95 and an average effect size of 0.15 (Faul et al., 2007). In total, 360 questionnaires were sent out, but only 238 responses were received. Out of 238 responses received, 201 are useable, as 37 responses were excluded because of incomplete or flawed responses making the response rate 56% (201/360). According to Comery and Lee (1992) inferential statistics approach, a sample of this size is a good sample.

### Measures

The situation allowed us to adapt or when possible use validated measures derived from extensive inquiry into the extent of literature. The items in each construct were measured using a five-point Likert scale. The first English version of the Questionnaire has 27 construct items in total. We used a professional translator to translate the first English version into Mandarin Chinese. The translated Mandarin version was back-translated into English. The translators and one researcher are both well-versed in English and Chinese. The back-translation was done to avoid potential discrepancies arising from the translation process. To ensure that questionnaire is clear and understandable in the Chinese automobile industry, we incorporated the feedback of two industry experts and two academicians. The Chinese version of the survey instrument was administered in the data collection process. The independent variable knowledge management capability process factors based on the 13-items scale were adopted from Lin and Lee (2005). Knowledge management capability process factors are established on three aspects: Knowledge acquisitions (four items), knowledge sharing (four items), and knowledge application (five items). The dependent variable firm-level new product development performance based on the five-items scale was adopted from Liu and Tsai (2007). To measure the mediating effect of organizational agility, the five-items scale was adopted from the study of Hoonsopon and Puriwat (2019). Lastly, the scale of moderating variable business model innovation measured by four items was derived from Huang et al. (2012). All the indicators were measured on a five-point Likert scale, 1 = Strongly disagree, 2 = Disagree, 3 = Undecided, 4 = Agree, and 5 = Strongly agree.

## Results

### Measurement model assessment

**Figure 2:**
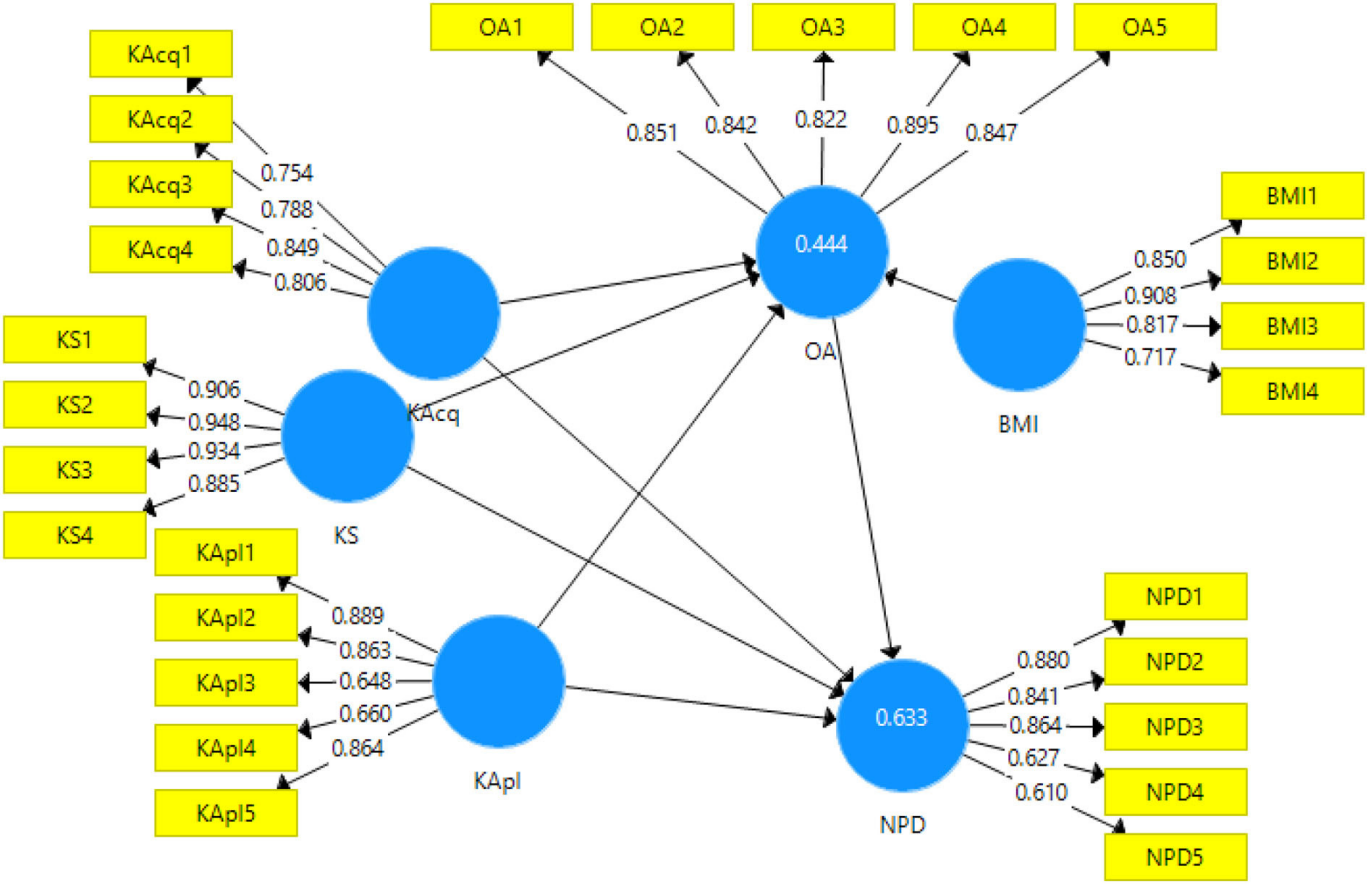
Measurement model assessment.

The measurement model was estimated in the partial least squares-structural equation model (PLS-SEM) using Smart PLS version 3.0 (Schlittgen et al., 2016). It was used to measure the inner consistency of constructs by factor loading, Cronbach's alpha, composite reliability (CR), and average extracted variance (AVE) (Henseler, 2017). Discriminant validity was also evaluated by using a measurement model. The results of the measurement model are shown in Figure 2 and Table 1.

**Table 1:** Measurement model.

**Construct**	**Items**	**Loadings**	**Cronbach's alpha**	**CR**	**AVE**
Business model innovation	BMI1	0.850	0.792	0.857	0.566
	BMI2	0.908			
	BMI3	0.817			
	BMI4	0.717			
Knowledge acquisition	KAcq1	0.754	0.812	0.876	0.640
	KAcq2	0.788			
	KAcq3	0.849			
	KAcq4	0.806			
Knowledge application	KApl1	0.889	0.844	0.892	0.627
	KApl2	0.863			
	KApl3	0.648			
	KApl4	0.660			
	KApl5	0.864			
Knowledge sharing	KS1	0.906	0.938	0.956	0.844
	KS2	0.934			
	KS3	0.948			
	KS4	0.885			
New product development	NPD1	0.880	0.824	0.879	0.598
	NPD2	0.841			
	NPD3	0.864			
	NPD4	0.627			
	NPD5	0.610			
Organization agility	OA1	0.851	0.905	0.929	0.725
	OA2	0.842			
	OA3	0.822			
	OA4	0.895			
	OA5	0.847			

Table 1 indicates alpha's value, composite reliability, and value of AVE. These values measure the convergent validity of the constructs, and all the values of this study meet the threshold level. According to the instructions of Gliem and Gliem (2003), Cronbach's alpha's value should be more than 0.6, and the value of Cronbach's alpha for all items of every variable is well above 0.6. Moreover, Fornell and Larcker (1981) recommended that the value of AVE should be more than or equal to 0.5, and the composite reliability value should be equal or 0.7 or above. The values of this study fall within the acceptable range of AVE and composite reliability.

Table 2 indicates the value of the HTMT ratio, which is another effective and alternative method to access the discriminant validity. Kline (2011) recommended an HTMT ratio of <0.85 to confirm the discriminant validity. Accordingly, all the values are higher than the benchmark level, which shows this study fulfills discriminant validity criteria.

**Table 2:** Heterotrait–Monotrait ratio (HTMT).

**Constructs**	**BMI**	**KAcq**	**KApI**	**KS**	**NPD**	**OA**
BMI						
KAcq	0.627					
KApI	0.628	0.807				
KS	0.557	0.788	0.716			
NPD	0.736	0.810	0.805	0.638		
OA	0.541	0.718	0.628	0.566	0.829	

*KApl, Knowledge Application; KAcq, Knowledge Acquisition; BMI, Business Model Innovation; NPD, New Product Development; OA, Organization Agility*.

### Structural model assessment

**Figure 3:**
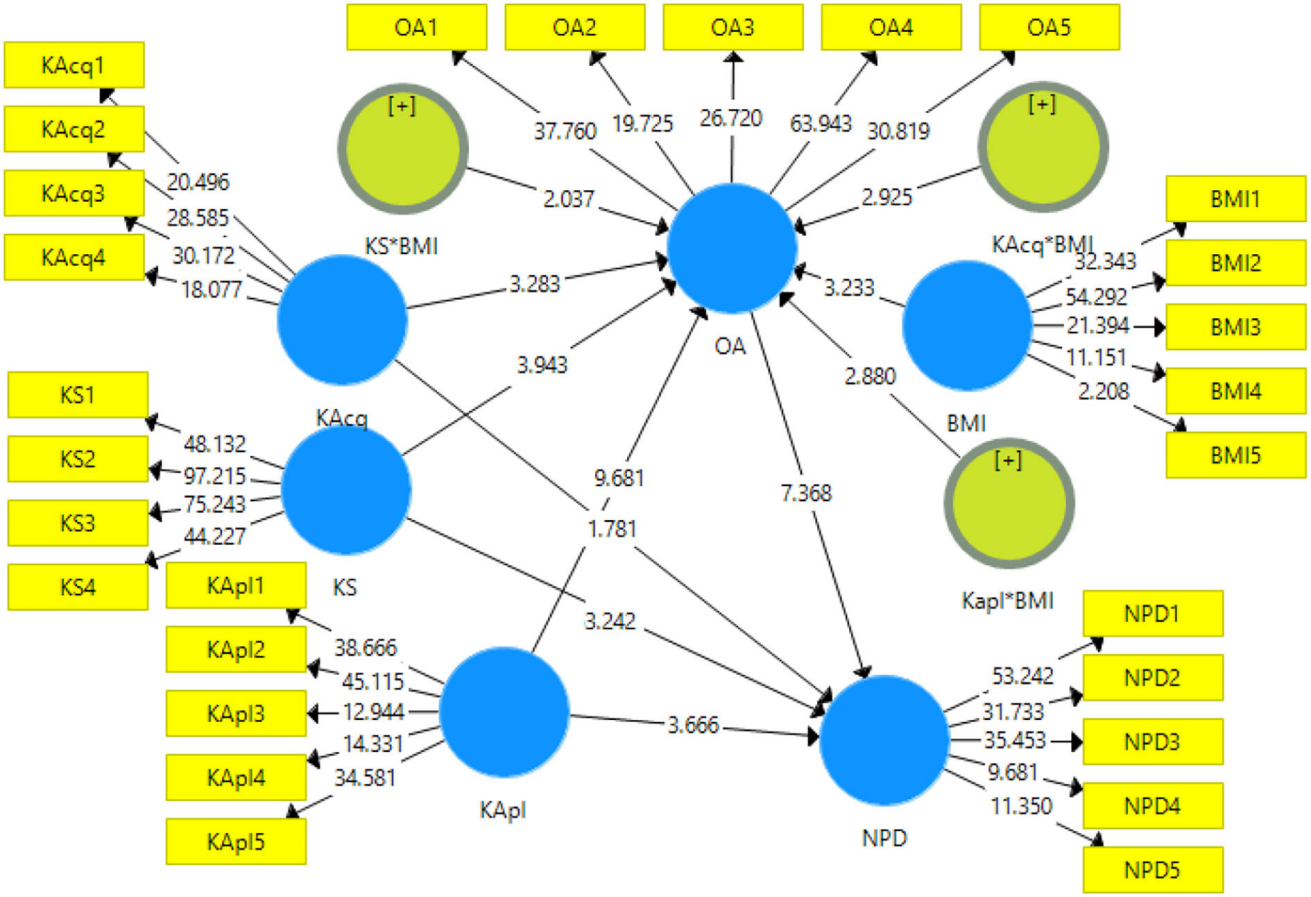
Structural model assessment (*n* = 5,000 bootstrapped samples).

To examine the relationship within the variables, a structural model analysis was conducted. This study adopts the bootstrapping method to examine the significance of path coefficients (see Figure 3). Results indicated that knowledge acquiring capability has no significant association with the performance of new product development. Therefore, H1 is not accepted. Moreover, results revealed that knowledge sharing is significantly and positively related to new product development performance (β = 0.272, *t* = 3.242), and H2 is supported. Moreover, knowledge application and organization agility are significantly and positively related to job new product development performance, and H3 and H4 are supported. The bootstrapping process specified the mediation effects. The findings show that organizational agility significantly and partially mediates the relationship between knowledge acquiring new product development performance (β = 0.169, *t* = 3.458), and H5 is supported. Results also indicate that organizational agility significantly and fully mediates the relationship between knowledge sharing and new product development performance (β = 0.105, *t* = 2.109), and H6 is supported. Results revealed that organizational agility significantly mediates the relationship between knowledge application and new product development performance (β = 0.087, *t* = 2.544), and H7 is supported.

Table 3 indicates the results of the moderation analysis. The findings revealed that business model innovation significantly and positively moderates the relationship of knowledge acquisition with organizational agility (β = 0.232, *t* = 2.909), and H8 is supported. Furthermore, business model innovation has a significant moderation effect on the relationship of knowledge sharing with organizational agility (β = 0.121, *t* = 2.037), and H9 is accepted. Results also indicated that business model innovation significantly moderated the relationship of knowledge application with organizational agility (β = 0.189, *t* = 2.880), and H10 is supported.

**Table 3:** Structural model assessment (direct effect results and decision).

**Hypotheses**	**Relationship among constructs**	**β**	**S. D**.	***T*** **-values**	***P*** **-values**	**Remarks**
**Direct effect**						
H1	KAcq -> NPD	0.166	0.093	1.781	0.071	NS
H2	KS -> NPD	0.272	0.084	3.242	0.000	S
H3	KApl -> NPD	0.238	0.065	3.666	0.001	S
H4	OA -> NPD	0.494	0.067	7.368	0.000	S
**Mediating effect**						
H5	KAcq -> OA -> NPD	0.169	0.049	3.458	0.001	S
H6	KS -> OA -> NPD	0.105	0.050	2.109	0.035	S
H7	KApl -> OA -> NPD	0.087	0.034	2.544	0.011	S
**Moderating effect**						
H8	KAcq*BMI -> OA	0.232	0.079	2.909	0.004	S
H9	KS*BMI -> OA	0.121	0.059	2.037	0.042	S
H10	KApl*BMI -> OA	0.189	0.066	2.880	0.004	S

*KApl, Knowledge Application; KAcq, Knowledge Acquisition; BMI, Business Model Innovation; NPD, New Product Development; OA, Organization Agility; S.D, Standard Deviation*.

## Discussion

The intention of this research is to examine the effect of knowledge management capabilities based on three categories knowledge acquisition, knowledge application, and knowledge sharing on new product development project performance with mediating role of organizational agility and moderating role of business model innovation on the relationship of KMC with organizational agility. The results revealed that knowledge application, and knowledge sharing both positively influence new product development. However, knowledge acquisition is insignificant. The results of current research are in line with the prior studies by Liu and Tsai (2007) and Yildirmaz et al. (2018), who argued that sharing of knowledge by employees with their colleagues and its application in firm results in direct benefits during new product development. Moreover, knowledge sharing, and its application play an important role in planning and forecasting new products. Furthermore, the results of this study indicate that organizational agility positively and significantly mediates the relationship between knowledge management capabilities and new product development. According to Hoonsopon and Puriwat (2019), organizational agility helps organizations develop better teams that discover the needs of customers by using knowledge management capabilities. It also helps in selecting the appropriate technology for new product development that creates value for customers and increases the sustainability and profitability of the product. Lastly, the findings of this study also revealed that business model innovation positively and significantly moderates the relationship of knowledge management capabilities with organization agility. According to the findings, essential knowledge management capabilities for automobile companies that enable them to innovate their business model include the ability to acquire new external knowledge, convert it so that it is ready to use, and finally apply it for new product development.

## Theoretical and practical implications

There are various ways in which the findings of this research theoretically support the literature. First, our research contributes to the growing body of literature on the internal factors that affect BMI. Moreover, Teece et al. (2016) and Clauss et al. (2019) indicated that previous research has focused on the ability to use and re-use resources in different ways, as well as on cultural values and a willingness to change. Therefore, we focus specifically on how the automobile sector's BMI is affected by their organizational KMC in this investigation. Internal facilitators have been hypothesized to have a positive impact on Business model innovation (BMI); however, no research has tested this explicitly (Foss and Saebi, 2017). Lastly, our empirical results support previous conceptual and case study-based research by directly correlating KMC to BMI. In terms of practical implications, current research offers significant implications for managers and policymakers in the automobile sector. The purpose of this study was to investigate the impact of organizational KMC strength on their NPD performance and explore how it enhances their ability to respond when an organization actively seeks to deliver value in innovative ways. Managers need to develop a modern competitive business environment for the implication of knowledge management capabilities. The research presented an all-inclusive view of KMC and its impact on NPD performance. Organizations need to leverage their knowledge-based capabilities and organizational agility to develop a knowledge-based environment. Moreover, organizations may improve the sustainability of their product and their overall performance. Managers may utilize knowledge-based resources to add value in the course of developing products.

## Limitations and future directions

There are always ways to improve a study, as no study is perfect. This study also has some limitations. This study focused only on the automobile industry, where applications of knowledge management capabilities in new product development can be investigated further. This study was conducted in a shorter time in a geographically constrained area. A larger sample can generate better results that can add to the insight and generalization aspect of the findings. The data collection for the study was done at one specific point in time; however, for future research, it will be of great use to analyze the companies at various times to see the impacts of KMC, NPD, OA, and BMI implementation. The respondents of the study were managers, Team leaders, engineers, and supervisors of the company; however, future research sampling should involve numerous respondents in a business rather than only the top management to increase the validity of the findings. Only one mediator and one moderator were able to be investigated for this research because of time constraints. In light of this, further study may improve the model and investigate other mediators like absorptive capacity and employee knowledge-sharing behavior. Future studies can also check other moderators, such as the organization's culture and technological complexity. From this point forward, there are a number of potential paths that researchers might choose further in their investigations.

## Data availability statement

The raw data supporting the conclusions of this article will be made available by the authors, without undue reservation.

## Author contributions

All authors listed have made a substantial, direct, and intellectual contribution to the work and approved it for publication.

## Funding

This study was supported by grants from the National Natural Science Foundation of China (72171197).

## Conflict of interest

The authors declare that the research was conducted in the absence of any commercial or financial relationships that could be construed as a potential conflict of interest.

## Publisher's note

All claims expressed in this article are solely those of the authors and do not necessarily represent those of their affiliated organizations, or those of the publisher, the editors and the reviewers. Any product that may be evaluated in this article, or claim that may be made by its manufacturer, is not guaranteed or endorsed by the publisher.
